# Declines in Pneumonia Hospitalizations of Children Aged <2 Years Associated with the Use of Pneumococcal Conjugate Vaccines — Tennessee, 1998–2012

**Published:** 2014-11-07

**Authors:** Marie R. Griffin, Edward Mitchel, Matthew R. Moore, Cynthia G. Whitney, Carlos G. Grijalva

**Affiliations:** 1 Department of Health Policy, Vanderbilt University School of Medicine; 2 Division of Bacterial Diseases, National Center for Immunization and Respiratory Diseases, CDC

The 7-valent pneumococcal conjugate vaccine (PCV7) was added to the U.S. infant immunization schedule in the year 2000. By 2009, PCV7 introduction was associated with a 43% decline in all-cause pneumonia among U.S. children aged <2 years (1). In 2010, a new 13-valent pneumococcal conjugate vaccine (PCV13) replaced PCV7 in the infant immunization schedule, expanding protection from seven to 13 pneumococcal serotypes. To examine changes in all-cause pneumonia hospitalizations among children aged <2 years after the switch to PCV13, Tennessee hospital discharge data for 1998–2012 were analyzed. By 2012, all-cause pneumonia hospitalizations in children aged <2 years had declined an additional 27%, relative to the PCV7 years. Pneumonia hospitalizations were estimated to be 4.1 per 1,000 population in 2012, a historically low rate that represents a 72% decline from the rate before PCV7 introduction. Tennessee children aged <2 years experienced about 1,300 fewer pneumonia hospitalizations annually in 2011 and 2012 than in the years before pneumococcal conjugate vaccine (PCV) use. These data attest to the powerful impact of the PCV program on pneumonia in Tennessee children. The observed trend likely represents a major decline in pneumococcal pneumonia, which should stimulate a reassessment of current causes and appropriate management of pneumonia in children.

*Streptococcus pneumoniae* is widely recognized as the primary bacterial pathogen causing community-acquired pneumonia in children (2). However, identifying the cause of pneumonia in individual cases is difficult (3). An overall reduction in pneumonia was an expected outcome of PCV7 vaccination because the major U.S. pre-licensure trial reported 30% efficacy against radiographically defined pneumonia (4). However, short term clinical trials might not predict effectiveness over time and outside of clinical trials, and no comparable efficacy trial has been performed for PCV13. To examine changes in rates of all-cause pneumonia among children aged <2 years after the switch to PCV13, Tennessee hospital discharge data for 1998–2012 were analyzed. The Tennessee Hospital Discharge Data System records data on hospitalizations and emergency department (ED) visits from all nonfederal hospitals in Tennessee. Tennessee’s population is about 6.5 million and includes 2% of the U.S. population. Tennessee’s Hospital Discharge Data System data from 1998 through 2012 were used to identify Tennessee residents aged <2 years with hospital admissions or ED visits for pneumonia. This age group was chosen because most children aged <2 years would have received PCV13 by 2012, and this age group experienced the earliest and steepest decline in pneumonia rates after PCV7 introduction (1,5). All-cause pneumonia hospitalizations were defined by *International Classification of Diseases, Ninth Revision, Clinical Modification* (ICD-9-CM) codes as a first-listed discharge diagnosis of pneumonia (480.xx–486.xx or 487.0) or by a first-listed discharge diagnosis of meningitis (321.xx, 013.0.x, 003.21, 036.0, 036.1, 047, 047.0, 047.1, 047.8, 047.9, 049.1, 053.0, 054.72, 072.1, 091.81, 094.2, 098.82, 100.81, 112.83, 114.2, 115.01, 115.11, 115.91, 130.0, 320, 320.0, 320.1, 320.2, 320.3, 320.7, 320.81, 320.82, 320.89, 320.8, 320.9, 322, 322.0, or 322.9), septicemia (038.1x, 038.4x, 003.1, 020.2, 022.3, 031.2, 036.2, 038, 038.0, 038.2, 038.3, 038.8, 038.9, 054.5, 785.52, 790.7, 995.91, or 995.92) or empyema (510.xx) and a pneumonia code in another diagnosis field. Codes considered specific for pneumococcal infections were 481.xx, 038.2, 041.2, or 320.1 (1,5,6). To explore whether changes in rates of all-cause pneumonia were specifically related to the vaccination programs, comparisons were made with changes in rates of ED visits and hospitalizations for the treatment of bone fractures (ICD-9-CM codes 800–829.xx).

Monthly annualized rates for hospitalizations and ED visits for all-cause pneumonia and fractures were obtained by multiplying monthly numbers of hospitalizations and ED visits by 365.25 divided by the number of days in each month, and dividing this result by the respective annual Tennessee population estimate for children aged <2 years from the U.S. Census Bureau. Rates were expressed as hospitalizations and ED visits per 1,000 children annually.

Three periods were defined: pre-PCV (January 1998–December 1999), PCV7 (January 2001–June 2009), and PCV13 (July 2010–December 2012) years. Two years were omitted from the analyses, calendar year 2000, when PCV7 was introduced, and July 2009–June 2010, which encompassed the atypical pandemic influenza period and the introduction of PCV13. Annualized monthly rates were modeled using negative binomial regression accounting for seasonal variation. Modelling rates over the three periods allowed estimation of linear trends in these three periods as well as comparison of rates in the PCV13 period to rates that would have been expected if trends in the PCV7 and pre-PCV7 periods, respectively, had not changed. Relative rates (RR) were used to compare study periods and calculate percentage changes from PCV7 years and pre-PCV years to PCV13 years ([1 − RR] × 100); annual rate differences between these periods were also calculated.

The annual number of hospitalizations for pneumonia of Tennessee children aged <2 years were >2,000 in 1998 and 1999, before PCV7 introduction, and declined to <1,000 by 2010 through 2012, after introduction of PCV13 (Table 1). Only ≤2% of all pneumonias were coded as pneumococcal, and these declined as well. The median length of stay was 3–4 days throughout this period; in-hospital deaths were uncommon but appeared to decline.

Monthly annualized pneumonia hospitalization rates for Tennessee children aged <2 years showed the typical seasonal pattern with increases during winter (Figure). Pneumonia hospitalization rates were fairly stable in the pre-PCV period, declined substantially after PCV7 introduction, and were lower yet after PCV13 introduction.

Annual pneumonia hospitalization rates in Tennessee children aged <2 years decreased from 14.5 to 4.1 per 1,000 from pre-PCV years to PCV13 years. Compared with PCV7 years, the rate after introduction of PCV13 was 27% lower, indicating 1.5 fewer hospitalizations per 1,000 children. The total decline after the years before PCV7 introduction was 72%, or 10.5 fewer hospitalizations per 1,000 children annually (Table 2). There was a corresponding 83% decline in pneumonia hospitalizations coded as pneumococcal. In this analysis, visits classified as observation stays were counted as ED visits, not hospitalizations. If observation stays were counted as hospitalizations, pneumonia hospitalization rates declined 64%, from 15.2 per 1,000 in the pre-PCV period to 5.6 per 1,000 in the PCV13 period. There were no statistically significant changes in pneumonia ED visit rates and no significant declines in ED visits or hospitalizations for fractures.

## Discussion

The decrease in pneumonia rates described in this report suggests substantial direct benefits of PCV13 use in the early years after its introduction. All-cause pneumonia hospitalizations in children aged <2 years in Tennessee declined 27% after introduction of PCV13 in 2010 and a total of 72% after the introduction of PCV7 into the routine childhood immunization schedule in 2000. Among Tennessee children aged <2 years, these rate reductions meant >1,300 fewer pneumonia hospitalizations annually compared with the years before introduction of PCVs. During the full 12 years after PCVs were introduced, approximately 11,000 fewer children were hospitalized with pneumonia than would have been expected based on rates in the pre-vaccine years.

Declines in pneumonia hospitalizations in children aged <2 years in Tennessee in the first 10 years after PCV7 introduction were similar to those previously reported for U.S. children overall. Pneumonia hospitalizations in U.S. children aged <2 years declined 43.2% (95% confidence interval = 34.9%–51.6%) during 2000–2009 (1), nearly identical to changes observed in Tennessee by 2009. Consistent declines in childhood pneumonia have also been observed in multiple countries where PCVs have been introduced (7–9).

These findings indicate that the expanded coverage of six additional serotypes with PCV13 has also expanded the effectiveness of the U.S. PCV program against pneumonia. However, no serotype information is available to quantify the vaccine effectiveness against individual serotypes.

The findings in this report are subject to at least three limitations. First, this is an ecologic study that evaluated the impact of the U.S. PCV program in Tennessee; individual level vaccination data were not examined. However, since 2008, coverage with ≥3 doses of PCV was >93% among young Tennessee children, with similar high vaccination coverage levels maintained since PCV13 introduction.* Second, other factors (e.g., changes in admission criteria) might have influenced the observed changes in pneumonia hospitalizations. Nevertheless, a planned analysis of hospitalizations for fractures revealed no systematic declines during the study years, indicating the observed declines were not part of a generalized reduction in hospital admissions. Furthermore, the 72% decline in all-cause pneumonia observed since PCV introduction was accompanied by an 83% decline in pneumonias with a specific pneumococcal code. In addition, disease severity as judged by length of stay and in-hospital mortality did not increase, and there was no compensatory increase in pneumonia ED visits, further supporting a lack of change in admission practices that might account for the observed trends. One previous study of changes in all-cause pneumonia since PCV13 introduction from a nationally representative U.S. private insurance inpatient discharge record database reported a 21% decline in all-cause pneumonia for children aged <2 years coincident with introduction of PCV13, which is similar to these findings in Tennessee (10). Finally, this study is restricted to the first 2 years after PCV13 introduction and although this assessment indicates an early impact on pneumonia hospitalizations, longer-term monitoring of changes in pneumonia incidence is warranted to obtain the full picture of vaccination effects.

What is already known on this topic?

Introduction of the 7-valent pneumococcal conjugate vaccine (PCV7) in 2000 was associated with a 43% decline in pneumonia hospitalizations in U.S. children aged <2 years by 2009.

What is added by this report?

Tennessee hospital discharge data documented a 27% decline in pneumonia hospitalizations in children aged <2 years by 2012, after the switch from PCV7 to 13-valent pneumococcal conjugate vaccine in 2010. The rate was estimated to be 4.1 per 1,000 population in 2012, a historically low rate that represents a 72% decline from the rate before PCV7 introduction in 2000. Tennessee children aged <2 years experienced about 1,300 fewer pneumonia hospitalizations annually in 2011 and 2012 than in the years before the use of pneumococcal conjugate vaccines (PCVs).

What are the implications for public health practice?

State health departments can use administrative data to evaluate the local impact of PCV vaccination programs. Decreases in pneumonia hospitalizations for children aged <2 years in Tennessee highlight the need to reassess current causes and appropriate management of childhood pneumonia.

Although the pneumococcus was reported to be responsible for 20%–60% of community-acquired pneumonias before PCV introduction (2), the proportion caused by serotypes included in PCVs was unknown. These findings suggest that in the pre-PCV era, a large proportion of childhood pneumonia hospitalizations were caused by the pneumococcal serotypes included in PCV13. The introduction of PCVs into the U.S. infant immunization schedule has resulted in a major change in the epidemiology of pneumonia in young children and, importantly, these vaccine-induced changes can be monitored using readily available, state-based hospital discharge data. These results are an incentive to maintain high vaccination coverage with PCVs. In addition, the causes and appropriate treatment of childhood pneumonia in the era of PCVs needs to be continually assessed because the distribution of bacterial and other causes of pneumonia will likely change.

## Figures and Tables

**FIGURE:**
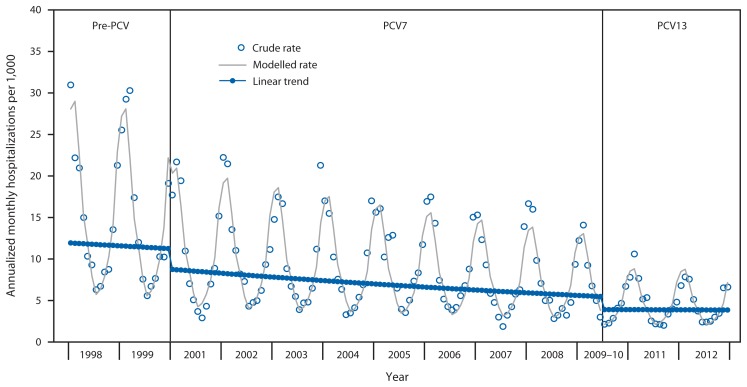
Annualized monthly all-cause pneumonia hospitalizations per 1,000 children aged <2 years during pre-pneumococcal conjugate vaccine (PCV), PCV7, and PCV13 years — Tennessee, 1998–2012 **Abbreviations:** PCV7 = 7-valent pneumococcal conjugate vaccine; PCV13 = 13-valent pneumococcal conjugate vaccine.

**TABLE 1:** Pneumonia hospitalizations for children aged <2 years, by selected characteristics — Tennessee, 1998–2012

Year	No. of pneumonia hospitalizations	No. with a pneumococcal code	Median stay (no. of days, interquartile range)	No. of in-hospital deaths
1998	2,047	44 (2.1)	4 (3,5)	4
1999	2,181	48 (2.2)	3 (3,5)	5
2000	1,744	32 (1.8)	3 (3,4)	2
2001	1,505	15 (1.0)	3 (3,4)	5
2002	1,518	10 (0.7)	3 (3,4)	3
2003	1,482	14 (0.9)	3 (3,5)	1
2004	1,306	12 (0.9)	3 (3,4)	4
2005	1,391	14 (1.0)	3 (3,4)	1
2006	1,364	18 (1.3)	3 (3,4)	2
2007	1,077	21 (1.9)	3 (3,4)	2
2008	1,114	17 (1.5)	3 (3,5)	0
2009	1,057	11 (1.0)	3 (3,4)	0
2010	829	17 (2.1)	4 (3,5)	1
2011	663	9 (1.4)	4 (3,5)	1
2012	673	8 (1.2)	3 (3,5)	1

**TABLE 2:** Annual hospitalizations and emergency department visits per 1,000 children aged <2 years for pneumonia and fractures during pre-pneumococcal conjugate vaccine (PCV), PCV7, and PCV13 years, and percentage change and rate differences comparing PCV13 years (July 2010–December 2012) with PCV7 years (January 2001–June 2010) and pre-PCV years (January 1998–December 1999)* — Tennessee, 1998–2012

	Annual events per 1,000 children aged <2 years			PCV13 years compared with PCV7 years†				PCV13 years compared with pre-PCV7 years†			
			
Condition	Pre-PCV years	PCV7 years	PCV13 years	% change in rates	(95% CI)	Rate difference per 1,000	(95% CI)	% change in rates	(95% CI)	Rate difference per 1,000	(95% CI)
**Pneumonia**											
Hospitalizations	14.5	8.6	4.1	−27	(−41 to −10)	−1.5	(−2.3 to −0.6)	−7.2	(−77 to −65)	−10.5	(−11.3 to −9.5)
ED visits	18.4	21.5	19.7	−8	(−21 to 7)	−1.8	(−4.6 to 1.5)	7	(−9 to 26)	1.3	(−1.6 to 4.7)
**Fractures**											
Hospitalizations	1.2	1.1	1.0	−15	(−35 to 11)	−0.2	(−0.4 to 0.1)	−12	(−33 to 16)	−0.1	(−0.4 to 0.2)
ED visits	5.5	6.1	6.4	0	(−11 to 12)	0	(−0.7 to 0.8)	17	(4 to 32)	1	(0.2 to 1.8)

**Abbreviations:** CI = confidence interval; PCV7 = 7-valent pneumococcal conjugate vaccine; PCV13 = 13-valent pneumococcal conjugate vaccine.

* The same calculations for fractures are included for comparison.

† Change in rates reflects changes in modeled trends and are not computed from rates displayed in columns 2–4.

## Footnotes

* Additional information available at http://www.cdc.gov/vaccines/imz-managers/coverage/nis/child/index.html.
